# The importance of sleep studies in improving the health indices of a nation

**DOI:** 10.1016/j.sleepx.2022.100049

**Published:** 2022-05-27

**Authors:** Jitendra Kumar Sinha, Kshitij Vashisth, Shampa Ghosh

**Affiliations:** GloNeuro, Sector 107, Vishwakarma Road, Noida, 201301, India; Amity Institute of Neuropsychology and Neurosciences (AINN), Amity University UP, Noida, 201303, India; Amity Institute of Neuropsychology and Neurosciences (AINN), Amity University UP, Noida, 201303, India; GloNeuro, Sector 107, Vishwakarma Road, Noida, 201301, India; ICMR – National Institute of Nutrition, Tarnaka, Hyderabad, 500007, India

Sleep quality has been considered as one of the most important parameters of good sleep health. A recent study [1] conducted on Singapore residents identified the different correlated (sociodemographic) factors of poor sleep outcome using the Pittsburg Sleep Quality Index (PSQI), which is a well accepted technique to study sleep in a large population [2]. The study links poor sleep with a marked decrease in health outcomes; importantly, metabolic syndrome, multimorbidity and mental health issues. It is also linked to unhealthy lifestyle practices such as smoking and excessive alcohol consumption.

The strength of the study lies in the inclusion of various ethnicities and groups across the country. Similar studies with regional modifications need to be performed in other countries to find out the modifiable factors that can help in improving quality of sleep as well as overall health of the population. Inclusion of sleep medicine specialists at district/county-level hospitals would further enhance the health indices significantly in the coming years.

The recent advancements in artificial intelligence based trackers and diagnostics bring a lot of hope for affordable sleep health improvement applications [3]. Ethical data collected through actigraphy and other affordable techniques could bring a massive change in understanding sleep health at global level. As a caution, we also need to be careful about the application of technology to certain groups vulnerable to sleep disorders like older population, people with mental disorders or other medical conditions. The psychological stress due to misdiagnosing someone with a sleep disorder should also be considered while informing the analytical results.
