# Erratum to “The importance of sleep studies in improving the health indices of a nation” [Sleep Med: X 4 (2022) 100049]

**DOI:** 10.1016/j.sleepx.2024.100130

**Published:** 2024-11-12

**Authors:** Jitendra Kumar Sinha, Kshitij Vashisth, Shampa Ghosh

**Affiliations:** a GloNeuro, Sector 107, Vishwakarma Road, Noida, 201301, India; b Amity Institute of Neuropsychology and Neurosciences (AINN), Amity University UP, Noida, 201303, India; c ICMR – National Institute of Nutrition, Tarnaka, Hyderabad, 500007, India

The publisher regrets “Declaration of conflict of interest” was not included in the published article that appeared in the previous issue of Sleep Medicine: X.

## Declaration of competing interest

The authors declare that they have no known competing financial interests or personal relationships that could have appeared to influence the work reported in this paper.

The publisher would like to apologise for any inconvenience caused.

